# Cranial anatomy of a Late Cretaceous aspidorhynchid fish (Neopterygii: Aspidorhynchiformes) from Alberta, Canada

**DOI:** 10.1002/ar.70114

**Published:** 2025-12-23

**Authors:** Mondo Miyazato, Luke E. Nelson, Joshua A. Doyon, Donald B. Brinkman, Alison M. Murray

**Affiliations:** ^1^ Department of Biological Sciences University of Alberta Edmonton Alberta Canada; ^2^ Royal Tyrrell Museum of Palaeontology Drumheller Alberta Canada

**Keywords:** Aspidorhynchiformes, Campanian, micro‐computed tomography, myodome, Teleostei

## Abstract

*Belonostomus longirostrisis* was named for an isolated jaw fragment from freshwater Upper Cretaceous (Campanian) sediments of the Dinosaur Park Formation of Alberta, Canada. Following the description of the Albertan species, numerous isolated cranial and postcranial elements have been collected from the Dinosaur Park Formation and assigned to *B. longirostris*, despite the material not overlapping with, and therefore not being comparable to, the holotype. This includes two partial skulls that lack jaws. These two skulls were assigned to the genus based on their clear aspidorhynchid affinities including ganoine‐covered dermal bones, and lack of other known taxa from the locality to which they could be ascribed. The two skulls were scanned with micro‐computed tomography (μ‐CT) to allow description of the cranial elements and their internal structure. The bones of the dermal skull roof are almost entirely fused together, ornamented with conspicuous ridges and tubercles, and bear a pair of supraorbital sensory canals along the lateral sides. Internally, many bones of the skull are fused together, with only a few elements that can be consistently isolated in the μ‐CT scans. Notable discoveries in this material include extensive enclosed cranial sensory canals, no fusion between the basioccipital and first vertebra, the anterior myodome restricted to the sphenotic region with complete lack of a posterior myodome, and a canal, presumably for the jugular vein, that does not connect with the trigemino‐facialis chamber. These last two features are important in that they would exclude Aspidorhynchidae from belonging to Teleostei.

**Abbreviations**
CMN: Canadian Museum of Nature, Ottawa, ON, Canada
TMP: Royal Tyrrell Museum of Palaeontology, Drumheller, AB, Canada

## 1 INTRODUCTION

Aspidorhynchiforms are Mesozoic neopterygian fishes found in all continents except Asia (Brito, 1997). These extinct fishes superficially resemble the extant gars (Lepisosteiformes) with their long, slender bodies, and posteriorly placed dorsal and anal fins. The fin placement indicates that they were likely ambush predators, which would have been able to generate a quick burst of propulsion with the posterior fins, but would not have sustained strong swimming ability. Similar to gars, aspidorhynchiforms bear ganoid scales, but they differ from gars in that the extended rostrum is formed by the dermethmoid (= rostral) and premaxillary bones (Brito, 1988) rather than premaxilla and lachrymo‐maxillary bones as in gars (Grande, 2010). Whether or not Aspidorhynchiformes belongs in Teleostei has been a matter of debate.

The Aspidorhynchidae is the only family within Aspidorhynchiformes. Species assigned to Aspidorhynchidae are placed in six genera: *Aspidorhynchus* Agassiz, 1833, *Belonostomus* Agassiz, 1834, *Vinctifer* Jordan, 1919, *Richmondichthys* Bartholomai, 2004, *Pseudovinctifer* Arratia, 2015, and *Jonoichthys* Gouiric‐Cavalli, 2015. Agassiz (1833) erected the genus *Aspidorhynchus* for two Late Jurassic fish with elongate toothed upper jaws projecting past the lower jaw from the marine Solnhofen limestones of Bavaria, Germany. Later, Agassiz (1834) removed one of these from *Aspidorhynchus* to his new genus *Belonostomus*, which he considered closely related, but distinct, based on several features, including that the upper and lower jaws of *Belonostomus* are of almost equal length, whereas the upper jaw of *Aspidorhynchus* is longer than the lower. However, the type species of *Belonostomus*, *Belonostomus tenuirostris* (Agassiz, 1833), was described as having the upper jaw longer than the lower (Agassiz, 1833) and specimens of this species, including the designated neotype (Ebert, 2014: fig. 2) show that the upper jaw extends significantly past the lower. At least some species, such as *B. helgolandicus* (Brito, 1997: fig. 50), have upper and lower jaws of comparable length. Brito (1997) provided an emended diagnosis for *Belonostomus* that included the predentary being of similar size or slightly smaller than the premaxilla, and presence of premaxillary teeth. This was supported by Ebert (2014), who noted in his diagnosis that it is the predentary and dentary bones of the lower jaw being of similar length in *Belonostomus* that distinguish the genus.

The genus *Vinctifer* was erected for *Aspidorhynchus comptoni* (Agassiz, 1841), which had previously been transferred to *Belonostomus* by Woodward (1890), and allied European species which Jordan (1919) noted had relatively short jaws (compared to the elongate jaws of *B. tenuirostris*), relatively robust body, and deep scales along the lateral body. *Richmondichthys* was erected for specimens from Australian Albian deposits that were formerly named *Belonostomus sweeti* as well as some identified only as *Aspidorhynchus* sp., with Bartholomai (2004) noting that this is a very large fish in which the lower jaw extends past the upper jaw. The holotype of *Pseudovinctifer* is an isolated lower jaw (Arratia, 2015), so relative upper and lower jaw lengths are unknown. Based on the above, the upper jaw is longer than the lower in *Aspidorhynchus*, and most but not all species of *Belonostomus*, but in the latter the jaws are longer in comparison to the body than in the former. In *Vinctifer*, the jaws are relatively short, the lower jaw is longer than the upper in *Richmondichthys*, and the upper jaw is unknown in *Pseudovinctifer*. *Jonoichthys*, known from the anterior portion of an articulated skeleton, has the upper jaw only slightly longer than the lower (Gouiric‐Cavalli, 2015). The relatively long jaws of *Belonostomus* have been used to identify aspidorhynchid fishes from fossil deposits of Alberta, Canada, as belonging to that genus.

A number of fragmentary long, thin aspidorhynchid jaw bones have been recovered from freshwater sediments of Campanian age of the Dinosaur Park Formation of southern Alberta, Canada. *Belonostomus longirostris* (Lambe, 1902), originally named as the type species of *Diphyodus*, was erected for a partial bone, 23 mm long, broken at both ends and bearing the bases of teeth (Figure 1). Lambe (1902:30) identified this fragment (specimen CMNFV 1367) as a part of an elongate toothed upper jaw formed by the completely fused left and right premaxillae, belonging to a fish with a long, slender snout. At the time of its description, in a preceding companion article, Osborn (1902) noted that relationships of Lambe's new taxon were problematic, although Lambe (1902) himself made no comment on relationships of the fossil. Jordan (1923), in his classification of fishes, gave this species (as *Diphyodus longirostris*) its own family Diphyodontidae in which he included (but misspelled) the genus *Stenoprotome* Hay, 1903 that Hay (1903) had named questionably in the family Belonostomidae. Neither Diphyodontidae nor Belonostomidae are currently in use. Lambe's (1902) species was later transferred to the genus *Belonostomus* by Estes (1964) who noted it resembled *Belonostomus cinctus* from the English Chalk, and classified the genus in Aspidorhynchidae. In the same paper, Estes (1964) referred material from the Lance Formation upper Maastrichtian, Wyoming) to the same species, including more complete jaw fragments (rostra and predentaries) and ganoid scales. Estes (1964) noted the jaw fragments and scales had a ganoine covering with many small “nutrient” pores, and the bone surface was ornamented with very fine ridges and grooves running lengthwise and subparallel to one another. He associated the scales with the jaw fragments in the same taxon based on the morphology of these elements as described in the literature from disarticulated material of *B. cinctus* Agassiz, 1837 from the Turonian of England and articulated material of *B. comptoni* (= *Vinctifer comptoni*) from the Cenomanian Santana Formation of Brazil. The referred material from the Lance Formation (Estes, 1964) is currently identified as *B. longirostris* despite many of the specimens (e.g., scales) being elements that are not preserved in the holotype (CMNFV 1367) from the Dinosaur Park Formation.

**FIGURE 1:**
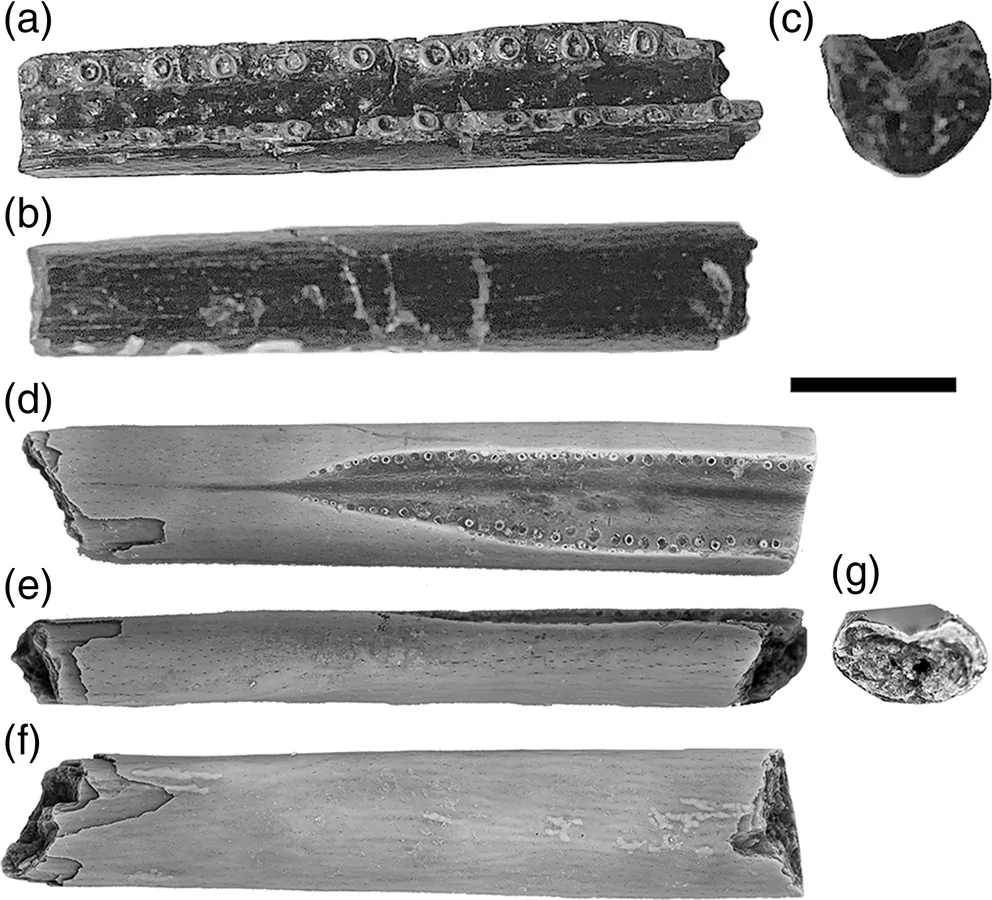
*Belonostomus longirostris* (Lambe, 1902) upper jaw fragments from Dinosaur Provincial Park, Alberta. (a–c) Holotype specimen CMNFV 1367, in (a), ventral, (b) dorsal, and (c) posterior views. (d–f) TMP 1964.004.102A in (d), ventral, (e) left lateral, (f) dorsal, and (g) anterior views showing the tooth rows that merge together and end before the anterior tip of the jaw. Anterior to left in dorsal, lateral, and ventral views. Scale bar equals 5 mm.

Three dimensionally preserved neurocrania of aspidorhychids are not well known. A partial neurocranium from Argentina was named the holotype of *Belonostomus lamarquensis*. That specimen preserves the posterior braincase and dermal bones (Bogan et al., 2011). *Jonoichthys challwa* is known from a more complete specimen preserved in three dimensions but details of the braincase are not clear (Gouiric‐Cavalli, 2015). Two neurocrania preserved in three dimensions are in the collections of the Royal Tyrrell Museum of Palaeontology. These were collected from the Dinosaur Park Formation and were cataloged as *B. longirostris*. Because *B. longirostris* is found in the same deposits, it is quite possible that the neurocrania belong to this taxon. The neurocrania can be confidently identified as belonging to an aspidorhynchid fish because of the fusion of the strongly ornamented dermal skull bones covered in ganoine; however, there is no anatomical overlap between these neurocrania and the holotype (partial upper jaw) of *B. longirostris*.

We here describe these two relatively complete skulls of *Belonostomus* from the Dinosaur Park Formation of southern Alberta, Canada, using micro‐computed tomography (μ‐CT) scanning. With this technique, we were able to isolate the specimen from the infilled matrix and observe the interior structure of the cranial elements. We document here the anatomy of the braincase and the sensory canals based on these two neurocrania and use this information to discuss the proposed relationship of Aspidorhychiformes to Teleostei.

## 2 MATERIALS AND METHODS

Different terms have been used to denote separate regions of the fish cranial skeleton by various researchers. Here, we use the term “neurocranium” to group the endochondral elements of the braincase with the attached dermal elements of the skull roof and parasphenoid. Terminology for the fused and individual cranial elements is similar to that used by Bogan et al. (2011) with composite terms used to indicate which bones we believe to be fused into single elements.

The two neurocrania described here were collected from the Dinosaur Park Formation from localities just outside the east boundaries of Dinosaur Provincial Park, Alberta, Canada. Specimen TMP 1985.036.349 (Figure 2) is a partial neurocranium preserving the posterior portion of the dermal skull roof, the bones of the braincase, and the posterior portion of the parasphenoid. It was collected from TMP locality L0014 (also called BB 14). Specimen TMP 1986.036.290 (Figure 3) is a less complete neurocranium that preserves the skull posterior to the region of the pterosphenoids and a fragment of the left pterosphenoid, but is missing the posterior portion of the neurocranium and one of the intercalars. It is from TMP locality L0268, also called Quarry 168. Both localities represent fluvial sandstones and the specimens were collected along with isolated dinosaur bones, champsosaurs, and other mesoreptile elements.

**FIGURE 2:**
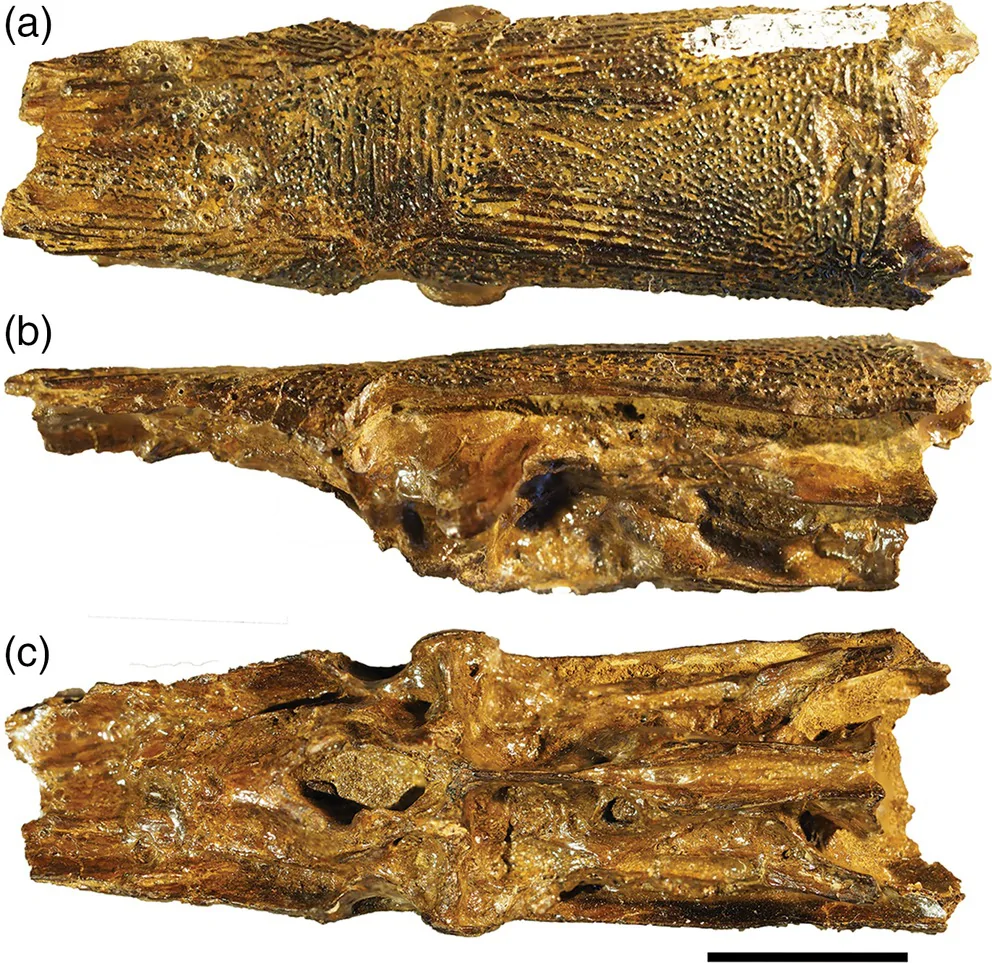
Photograph of specimen TMP 1985.036.349, a partial neurocranium, in (a) dorsal, (b) left lateral, and (c) ventral views. Anterior to left. Scale bar equals 1 cm.

**FIGURE 3:**
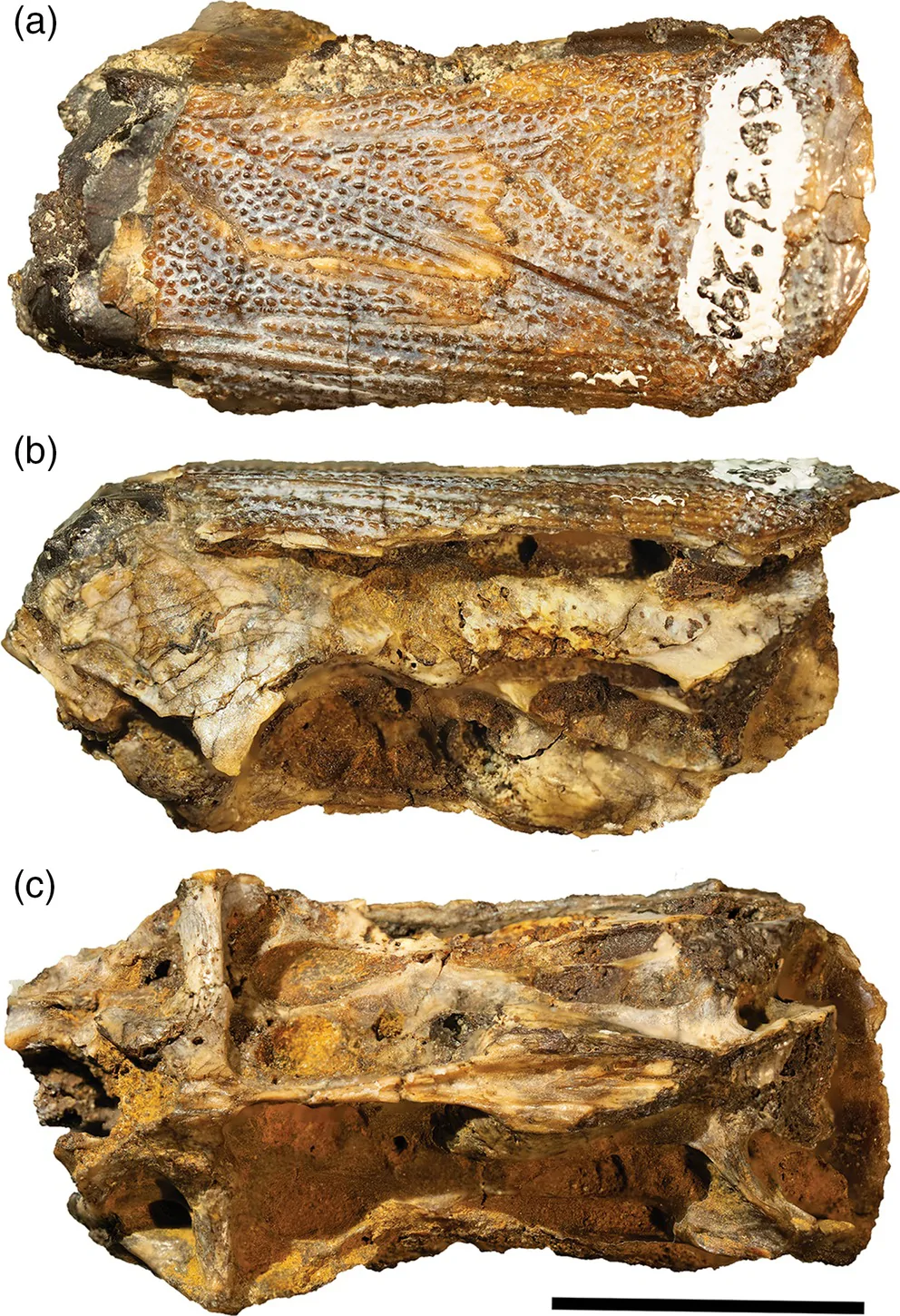
Photograph of specimen TMP 1986.036.290, a partial neurocranium, in (a) dorsal, (b) left lateral, and (c) ventral views. Anterior to left. Scale bar equals 1 cm.

Both fish neurocrania were scanned using a Nikon XTH‐225 ST micro‐CT scanner and the scan data was manipulated in Dragonfly, Version 2024.1 for Windows. Cranial elements and matrix were highlighted and segmented within the program based on density and structure. The highlighted cranial elements were then identified by comparison with materials described by Bogan et al. (2011) and Bartholomai (2004), as well as reference to modern fish neurocrania in the collections of the University of Alberta Museum of Zoology.

## 3 DESCRIPTION

The two neurocrania come from fish of roughly the same size. The length of the living fish is difficult to estimate, because the length of the rostrum is unknown; however, if the neurocrania come from fish with similar proportions to *B. tenuirostris*, then these individuals would have been about 26 and 36 cm in total length. The description provided here is based primarily on the larger, more complete specimen, TMP 1985.036.349 (Figure 2). We noted no significant differences in morphology between that specimen and TMP 1986.036.290 (Figure 3). There is significant fusion of the dermal skull bones and fusion of the endochondral braincase, but the bones of the orbital region are not fused, indicating that these were sub‐adult to adult individuals.

### 3.1 Dermal skull roof

The entire dermal skull roof of specimen TMP 1985.036.349 (Figure 4) is relatively flat, curving slightly ventrally on the lateral edges and tapering slightly anteriorly over the orbit. The preserved skull roof is formed by large left and right fused elements that are ornamented and covered in ganoine (Figures 2 and 3). Neither specimen shows a separate pterotic‐supratemporal, as identified by Bogan et al. (2011) in *Belonostomus lamarqensis*, or a separate extrascapular, as identified in *Aspidorhynchus sanzenbacheri* by Brito and Ebert (2008). There is also no separate dermosphenotic. We here identify the two elements of the skull roof as the left and right fused frontal + parietal + dermosphenotic + dermopterotic + extrascapular. The median suture between the left and right halves of the skull roof is sinuous, with broad, deep interdigitations. It is best identified consistently, both externally on the fossil and internally in the μ‐CT scan, in the smaller specimen, TMP 1986.36.290 (Figure 4). In the larger specimen (TMP 1985.036.349), the suture is clear posteriorly but becomes difficult to define in the orbital region. The skull roof is heavily ornamented with ridges and tubercles arranged roughly longitudinally, although some rows are more sinuous or irregular. The dermal bone of the skull roof appears uniform in structure internally, with occasional fractures that are likely taphonomic.

**FIGURE 4:**
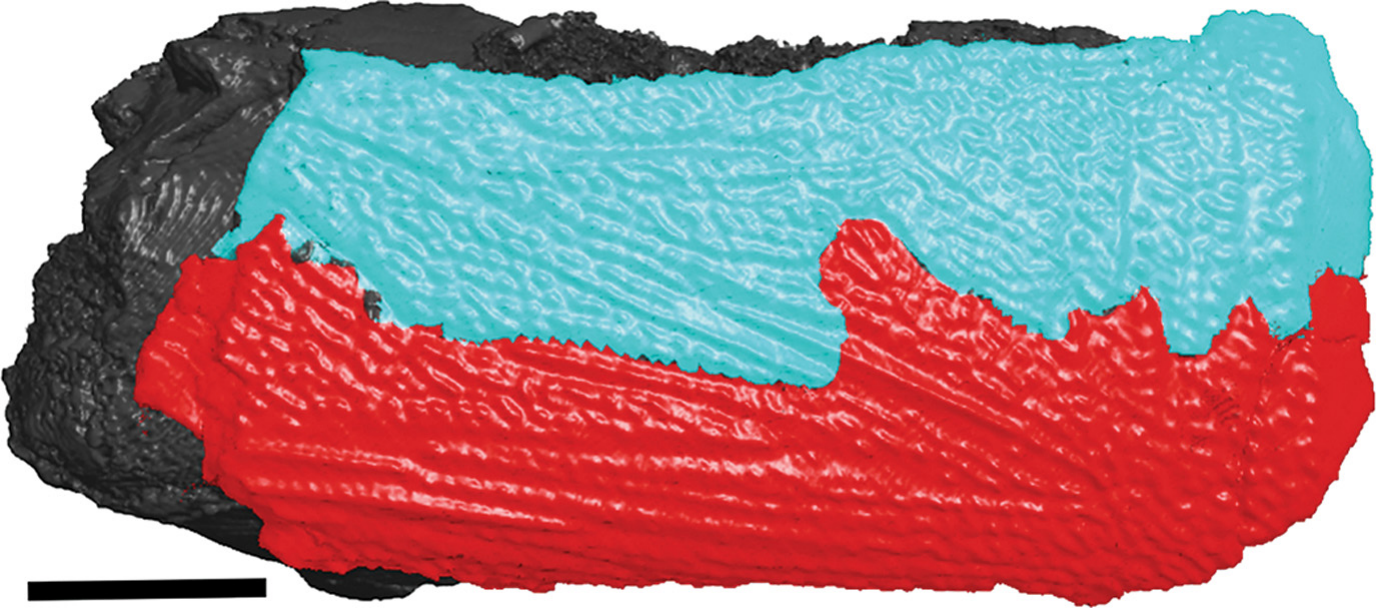
Rendering of the micro‐computed tomography scan of specimen TMP 1986.036.290, a partial neurocranium, in dorsal view with the right fused frontal + parietal + dermosphenotic + dermopterotic + extrascapular colored light blue, and the left element colored red. Anterior to left. Image taken from Dragonfly software. Scale bar equals 5 mm.

### 3.2 Orbital region

The median, unpaired orbitosphenoid is present anteriorly articulating with the ventral surface of the dermal skull roof in specimen TMP 1985.36.349 (Figure 5b), but this area is missing in TMP 1986.36.290. The orbitosphenoid forms the ventral floor that cradles a fairly large central opening that is wider posteriorly and narrows to a much smaller anterior opening (Figure 5c); this would have given passage to the olfactory nerves (CN I) passing from the brain to the nasal sacs. The posterior margin of the orbitosphenoid forms the anterior edge of a large median ventral opening which is identified as the opening for the optic nerves (CN II) from the brain. On either side of the median posterior opening, the orbitosphenoid meets the left and right pterosphenoids in a smooth transverse suture. Internally, the endochondral bone of the orbitosphenoid is porous, contains many open spaces, and appears similar to the cancellous bone as defined and identified in the centra of salmon (De Clercq et al., 2017). This same cancellous‐type bone is found in all the endochondral bones of the fossil neurocrania. There is no interorbital septum and no evidence that one would have been present because the bone edges are smooth, lacking any sign of breakage.

**FIGURE 5:**
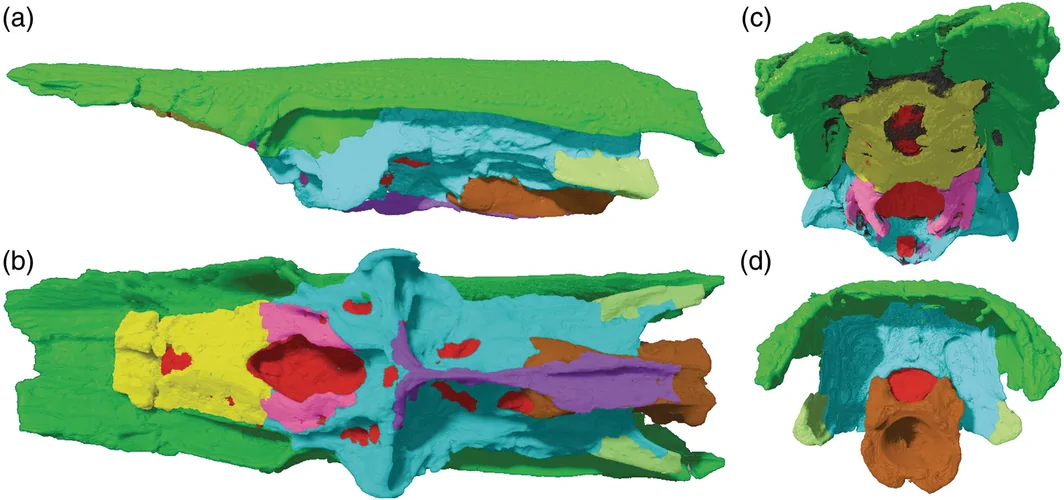
Rendering of the micro‐computed tomography scan of specimen TMP 1985.36.349 in (a) left lateral; (b) ventral; (c) anterior; and (d) posterior views. Colored elements are: bright green—fused frontal + parietal + dermosphenotic + dermopterotic + extrascapular; light blue—brain case formed of the fused sphenotic, prootic, pterotic, supraoccipital, and epioccipital; light green—intercalar; yellow—orbitosphenoid; pink—pterosphenoid; purple—parasphenoid; brown—fused basioccipital and exoccipitals; red—internal cranial cavity and nerve foramina. All images are screen captures from Dragonfly software. Scale bars equal 5 mm.

The pterosphenoids taper posteriorly (Figure 5b) and protrude medioventrally (Figure 5c), constricting the antero‐ventral opening in the brain case. The morphology of the paired pterosphenoids where they abut the posterior end of the orbitosphenoid and define the lateral edges of this opening for the optic nerves causes the opening to have an arrow‐head shape in ventral view. The sutures between the pterosphenoids and neighboring elements are only clear on the surface and in the bone immediately under it; the extensive porosity of the bone internally obscures the sutures between the elements to the extent that they cannot be determined in the μ‐CT scans. Three pairs of foramina pierce the lateral walls of the pterosphenoids, each separate from the others. There is no basisphenoid present; however, this likely was lost along with the anterior portion of the parasphenoid below the orbit.

### 3.3 Sphenotic region

The dermal skull roof prominently overhangs the braincase laterally along the length of the neurocranium, reaching its ventralmost point at the posterior edge of the orbit—this would be the area of the fused dermosphenotic. In this area, there is some separation between the frontal region of the fused dorsal skull roof element and the underlying autosphenotic, but these two elements appear fused for the most part, with no separation discernable in the μ‐CT scan between the underside of the frontal and the dorsal limit of the autosphenotic (Figure 5a). Although the autosphenotic can be partially distinguished from the prootic region of the fused braincase, the dermosphenotic is indistinguishably fused with the dermal skull roof and the distinction between the two parts of the sphenoid is not fully clear. In cross‐section in the μ‐CT scans, there is a distinct sinuous suture between the compact and cancellous bone in this area, which appears to indicate the separation of the dermosphenotic from the autosphenotic. This suture disappears more posteriorly.

### 3.4 Otic region

Most of the bones of the otic region (Figure 5) cannot be separated from one another and here, are identified as a single, unpaired braincase element that would be a fusion of left and right prootics and autopterotics, as well as the supraoccipital and epioccipitals. This fused element may also contain part of the exoccipitals as well, but this cannot be definitively determined. The prootic region abuts and curves around the lateral and posteroventral margins of the pterosphenoids anteriorly, and forms the ventralmost extent of the anterior opening in the braincase for the optic nerves. Dorsolaterally, the bone of the otic region is thin where it meets the autosphenotic extending ventrally from the underside of the dermal skull. In ventral view, the braincase is broadest just posterior to the opening for the optic nerves, at the level of the ascending processes of the broken parasphenoid. The lateral sides in this region extend ventrally in a slightly hooked wing‐like process. Foramina in this area are described separately below.

Ventrally, the otic region of the braincase meets the parasphenoid in the midline anteriorly and the basioccipital posteriorly (Figure 5b). The cranial cavity is laterally compressed in this region into a very thin ventromedial ridge extending anteriorly‐posteriorly that meets the parasphenoid ventrally. The braincase is wider just anterior to the anterior extent of the basioccipital, and two pairs of longitudinal ridges form on the ventrolateral surface of this region (Figure 5a). These ridges continue posteriorly along the lateral side of the braincase to reach the intercalar.

### 3.5 Occipital region

Fusion of bones also occurs in the occipital region. The basioccipital is extensively fused with the left and right exoccipitals so we refer to it here as a basi‐exoccipital (Figure 5d). This element is dominated internally by cancellous bone making the limits of the basioccipital and exoccipitals impossible to determine in the μ‐CT scans. The anterior end of the basioccipital portion of the element forms the ventrolateral walls of the cranial cavity medially. In ventral view, the basioccipital portion of the bone protrudes posteriorly past the posterior end of the parasphenoid. Dorsally, the basi‐exoccipital forms the ventral concave surface of the foramen magnum. The supraoccipital is fused with the bones of the braincase (prootic, etc.); however, in posterior view (Figure 5d), the small supraoccipital crest can be identified forming the roof of the foramen magnum and contacting the exoccipital region on either side. The first vertebra is not fused to the posterior basioccipital facet, and is not preserved in either specimen.

The intercalars are separate (unfused) elements (Figure 5a,b,d) at the posterolateral edge of the braincase. The identification of these bones as intercalars follows Brito (1992) and Grande and Bemis (1998) among others. The paired intercalars are approximately half as long as the basi‐exoccipital. In lateral view (Figure 5a), they are roughly rectangular and project posterolaterally. In cross‐section, they are pointed ventrolaterally, and are composed mostly of compact bone.

The posttemporal fossa is a large embayment in the dermopterotic region with the dermal skull roof curving ventrally along the lateral edge to roof the fossa dorsally and laterally (Figure 5a,d). The fossa extends far anteriorly into the sphenotic region.

### 3.6 Parasphenoid

The parasphenoid is broken, with only the portion ventral to the braincase and basioccipital preserved (Figure 5a,b). At the anterior end of the preserved portion, the parasphenoid expands laterally into the ascending processes which form a V‐shaped cross‐section. Posterior to this, the parasphenoid bears a sharp dorsal ridge that meets the medioventral ridge of the prootic region of the braincase. This area of the parasphenoid is very thin and small gaps appear in the bone because of the lack of resolution of the μ‐CT scan (Figure 5a). The parasphenoid then expands laterally as it meets the basi‐exoccipital, and forms a broad, shallow trough opening ventrally, where it underlies the basi‐exoccipital. Internally, the parasphenoid is very thin and composed of compact bone. Posteriorly, the parasphenoid ends prior to the posteriormost edge of the basi‐exoccipital.

### 3.7 Sensory canal system

A sensory canal extends from the anterior to posterior extremity of the preserved portion of the skull roof on either side, close to the lateral edge (Figure 6). The canal is contained within the bone, and no canal pore openings are visible; the canal would not have been apparent on the fossil without knowledge of its position based on the μ‐CT scan. An internal branch of the supraorbital sensory canal extends ventrally behind the orbit into the bone representing the dermosphenotic region of the fused dermal skull roof. Toward the posterior end, the canal has a shallow ventral dip; this appears to be where the fusion between the pterotic‐supratemporal and fronto‐parietal would be. Multiple smaller canals are visible radiating from the main canal, similar to the condition in *Vinctifer* noted by Brito (1997).

**FIGURE 6:**
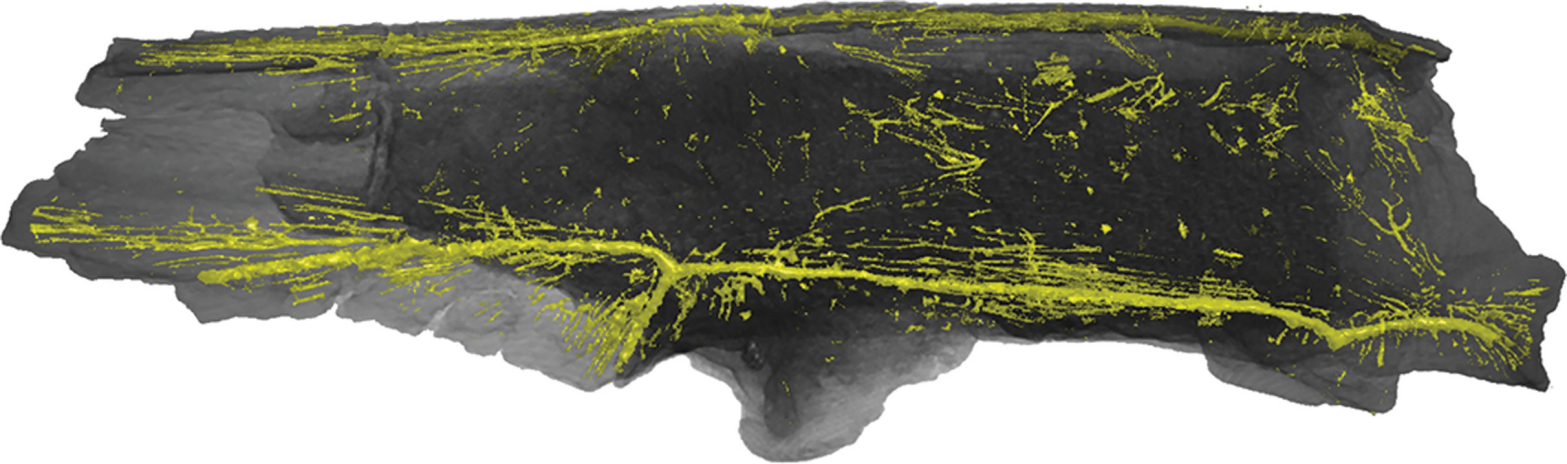
Rendering of the micro‐computed tomography scan of specimen TMP 1985.36.349 in dorsolateral view with the extent of the sensory canal highlighted in yellow. Anterior to left. Image is a screen capture from Dragonfly software.

### 3.8 Cranial openings and foramina

There are numerous larger openings and smaller foramina piercing the bones of the skull, most of which connect to the cranial space (Figures 7 and 8). We have identified them here by comparing their positions with the foramina indicated by other authors (e.g., Bogan et al., 2011; Brito, 1992; Patterson, 1964), the anatomy of extant fish brains, the anatomy of dried skeletal material of extant fishes, and by tracing their routes through the bones of the Dinosaur Park crania in the μ‐CT scan. We also note that some spaces we can see and trace in the μ‐CT scan might have been subdivided or otherwise enclosed with tissues (dura mater, connective tissue, or other) that are not preserved in the fossil.

**FIGURE 7:**
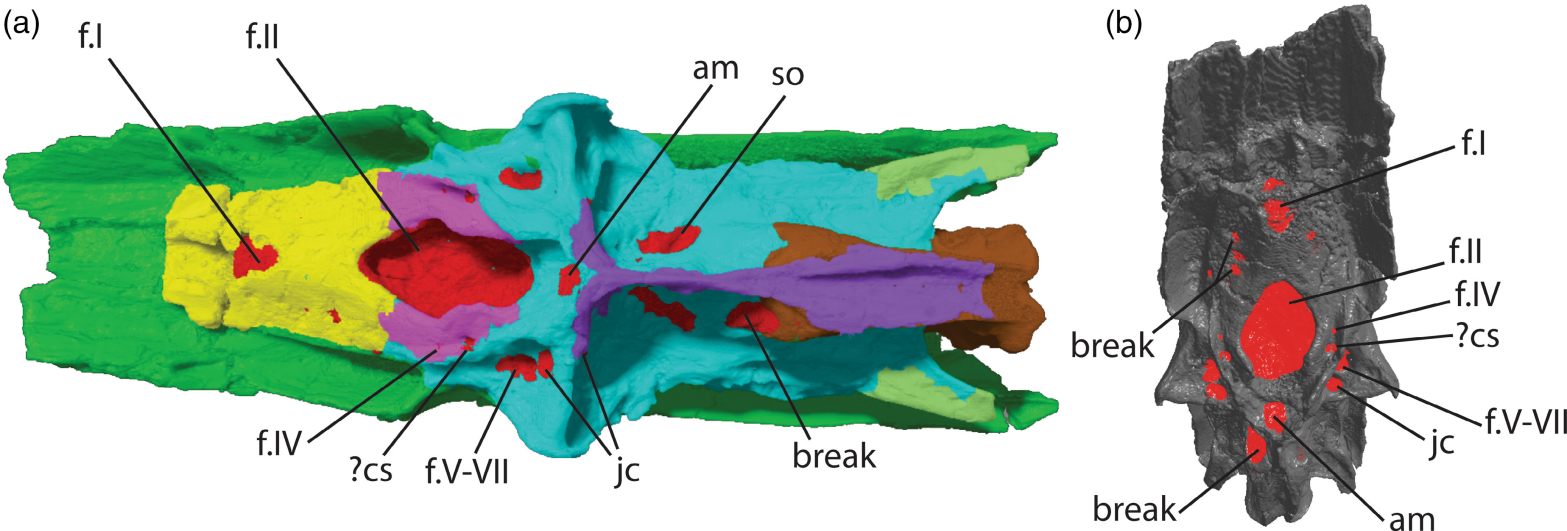
Rendering of the micro‐computed tomography scan of specimen TMP 1985.36.349 with the presumed identifications of the foramina noted. (a) Ventral view, anterior to left; (b) antero‐ventral view. am, opening of the anterior myodome; break, this is an area of thin bone, not an opening, that is an artifact of resolution in the micro‐computed tomography scan; ?cs, probably an opening for a vessel of the circulatory system; f.I, opening for cranial nerve I (olfactory nerve); f.II, opening for cranial nerve II (optic nerve); f.IV, opening for cranial nerve IV (trochlear nerve); f.V–VII, opening of trigemino‐facialis chamber for cranial nerves V (trigeminal) and VII (facial); jc, anterior and posterior opening of the canal for the jugular vein (this channel does not connect with the brain cavity and there are no connections between it and the trigemino‐facialis chamber); so, openings in the region of the saccular otoliths (these openings were not ossified but would presumably be closed in the living individual with a membrane). All images are screen captures from Dragonfly software.

**FIGURE 8:**
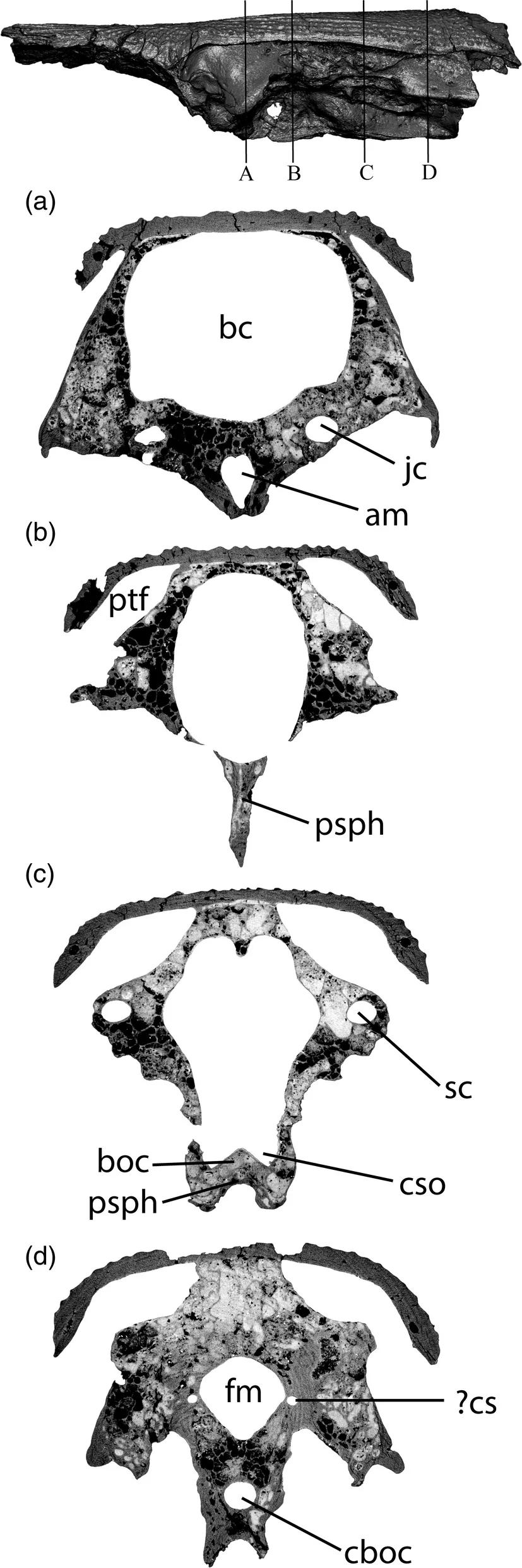
Cross‐sections through the neurocranium of TMP 1985.36.349 to show the extent of the myodome. Top, left lateral view of the skull showing the position of the four cross‐sections labeled (a–d). (a) Slice through the sphenotic region showing the anterior myodome; (b) slice through the prootic region anterior to the basioccipital showing no openings below the braincase; (c) slice through the anterior part of the basioccipital showing no ventral opening (i.e., no posterior myodome); (d) slice through the posterior part of the basioccipital. am, anterior myodome; bc, cavity for the brain; boc, basioccipital; cboc, concavity of the posterior facet of the basioccipital that appears as empty space in the cross‐section; ?cs, foramen for presumed passage of vessel of the circulatory system; cso, chamber for the saccular otolith; fm, foramen magnum; jc, canal presumably for the jugular vein; psph, parasphenoid; ptf, posttemporal fossa; sc, semicircular canal.

Anteriorly, the cranial space opens through a single median opening formed by the dermal skull roof and the orbitosphenoid to allow passage of the olfactory nerves (CN 1) anteriorly. Several other small openings in the orbitosphenoid posterior to this are taphonomic breaks (Figure 7b). The posterior edge of the orbitosphenoid and the lateral edges of the pterosphenoids define the large opening for the passage of the optic nerves (CN II) and the hypophysis more ventrally. It is possible that the oculomotor nerve (CN III) also passed through this opening. Piercing the bone of the each pterosphenoid are three small foramina (Figure 7a,b); the anterior one is identified as the foramen for the trochlear nerve (CN IV), and just posterior to that are two openings very close to one another, presumably for the circulatory system.

Two pairs of large foramina open in the anterior wall of the prootic region. The more anterodorsal pair connect with the cranial cavity and are identified as the openings of the trigemino‐facialis chamber that accommodated the trigeminal (CN V) and facial (CN VII) ganglia and nerves. Almost immediately posteroventral to these foramina, a second pair penetrate the wing‐like processes of the lateral skull but do not connect with the cranial cavity (Figure 8a); each exits posteriorly through a smaller foramen just dorsolateral to the parasphenoid (Figure 7a). These canals likely accommodated the jugular vein. There is no connection between this canal and the trigemino‐facialis chamber, unlike the condition in *V. comptoni* (Brito, 1992), indicating that the branches of the facial and trigeminal nerves could not have exited through this canal. This interpretation indicates that there was no pars jugularis in the trigemino‐facialis chamber, with the jugular vein accommodated separately (so we identify it as the jugular canal), perhaps with several nerves sharing the canal similar to the condition found in *Amia* (Grande & Bemis, 1998).

A large, single, median foramen opens anteriorly in the braincase wall just in front of the ascending processes of the parasphenoid (Figures 7a,b and 8a). This opening leads to an enclosed chamber that we identify as the anterior myodome. Although the rendering of the μ‐CT scan of the cranial space (Figure 9) indicates a significant connection between this chamber and the cranial cavity, this apparent connection is extremely narrow, restricted to the midline and would presumably have been closed over by the dura mater or a membrane that is not preserved in the fossil. Therefore, the apparent connection is an artifact of the fossil preservation and the scanning process. There is no evidence of a posterior myodome. There is no extension of the anterior myodome under the braincase (Figure 8b), and no ventral channel in the basioccipital dorsal to the parasphenoid (Figure 8c).

**FIGURE 9:**
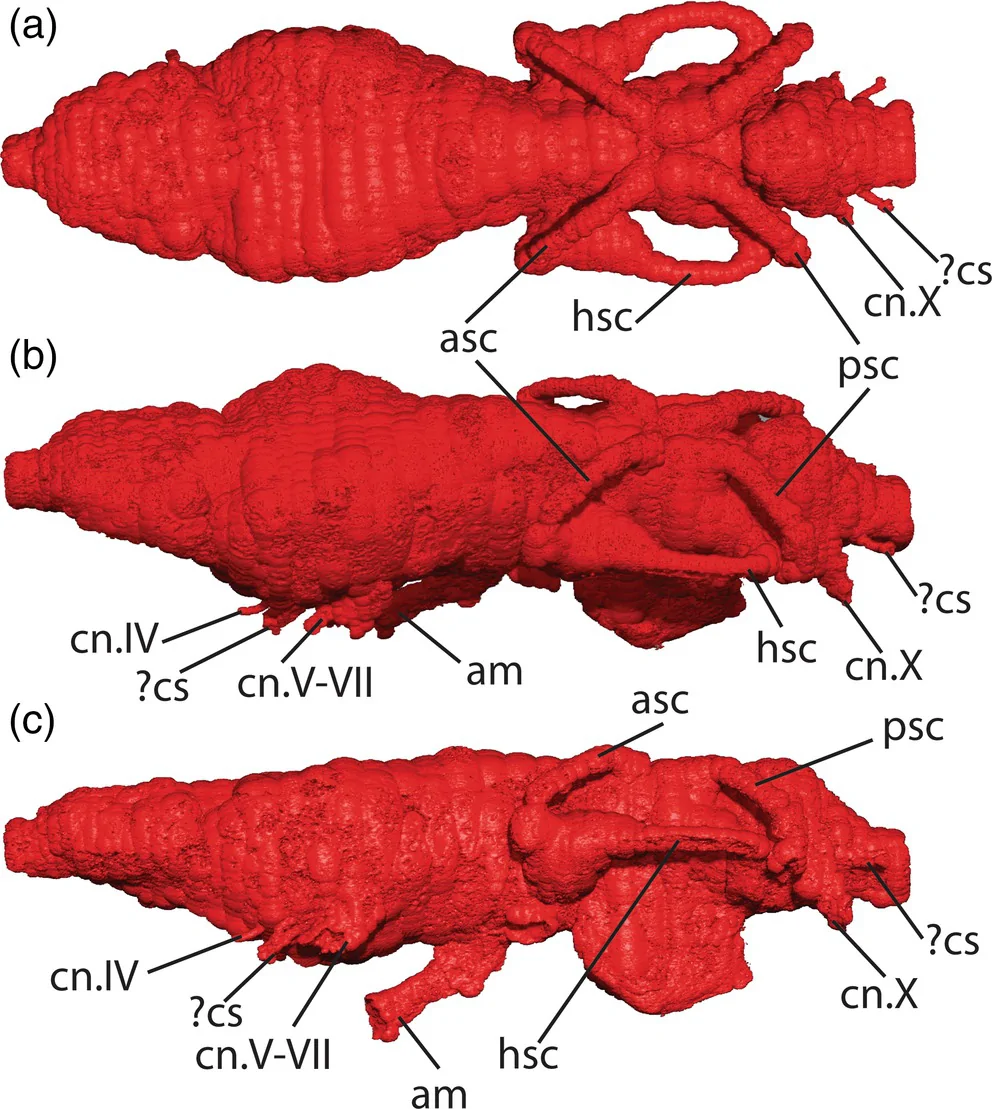
Endocast of the cranial cavity of TMP 1985.36.349 in (a) dorsal; (b) dorsolateral; and (c) left lateral views. All images are screen captures from Dragonfly software. The apparent connection between the anterior myodome and the brain cavity is an artifact of the micro‐computed tomography‐scan (see text). The ridged appearance of the endocast is not natural; it is an artifact of highlighting the skull elements in cross‐sectional view and the brush size (resolution) used in the software. am, anterior myodome; asc, anterior semicircular canal; ?cs, presumed vessel of the circulatory system; cn.IV, cranial nerve IV (trochlear); cn.V–VII, cranial nerves V (trigeminal) and VII (facial); cnX, cranial nerve X (vagus); hsc, horizontal semicircular canal; psc, posterior semicircular canal.

Another pair of large, oval openings in the ventral wall of the cranial cavity are situated in the otic region just ventral to the semicircular canals. These would likely have been closed by a membrane in life, which would have floored the chambers for the saccular otoliths.

The routes of exit from the braincase for cranial nerves VI, VIII, and IX (abducens, acusticovestibular or vestibulocochlear, and glossopharyngeal nerves) cannot been determined, but it is possible that one or more of those may also have been accommodated in the trigemino‐facialis chamber or the canal we identified for the jugular vein, or the foramina we suggest were for the circulatory system were instead for these nerves. Posteriorly, a small pair of foramina exit the braincase lateral to the basi‐exoccipital (Figure 8d). These match the position of the foramina for the vagus nerve (CN X) in a shark chondrocranium as well as in neopterygians (e.g., López‐Arbarello & Sferco, 2018); however, the endocast (Figure 9) shows a very small vessel passes through that foramen, and a larger one that we identify as the vagus nerve is located more anteriorly. We suggest these foramina are for vessels of the circulatory system.

### 3.9 Endocast and semicircular canals

The endocast (Figure 9) does not reflect the exact shape of the brain, because we cannot distinguish the left and right optic lobes from one another. Also, as noted by Brito (1992), the cranial space is unlikely to reflect the true shape of the brain. However, the overall size of the brain is likely similar to that of the endocast, particularly in the posterior region including the space for the medulla oblongata. The space within the bones of the braincase shows the three pairs of semicircular canals along with the fore, mid, and hindbrain, and overall, the endocast is generally similar to that of *V. comptoni* (Brito, 1992: fig. 6). A ventral projection of the Albertan endocast represents the anterior myodome, showing a connection that would likely have not been present in life, with a membrane separating the myodome from the brain (as noted above). The anterior myodome is a median structure as in *V. comptoni* (Brito, 1992). It is a simple, relatively short canal that angles posterodorsally and is limited to the prootic region of the skull. The roots of several nerves are apparent, as well as a few other vessels that we suggest are part of the circulatory system.

## 4 DISCUSSION

### 4.1 Comparison of Albertan crania to that of other aspidorhynchids

Cranial material from several aspidorhynchids has been described previously and distinctive fusion of elements in the skulls of these fishes has been documented. The suturing of the frontal, parietal, and dermopterotic bones with the suture between left and right sides becoming indistinguishable in larger (presumably older) individuals, and fusion of the endocranial bones has been reported for numerous aspidorhynchids (e.g., Bartholomai, 2004; Bogan et al., 2011; Brito, 1992, 1997; Brito & Ebert, 2008). The μ‐CT scans of the material presented here confirms these prior observations and indicates that, at least in the Albertan taxon, the left and right fused dorsal skull roof elements include the sphenotic and dermopterotic along with the frontal and parietal. Similarly, the left and right prootics and autopterotics are indistinguishably fused into a single element, which also includes the supraoccipital and epioccipitals. Fusion is also evident in the occipital region, with the left and right exoccipitals and basioccipital forming a single element. However, fusion of bones is not evident anteriorly, in the orbital region; the pterosphenoids and orbitosphenoid are distinct elements. This pattern of fused and unfused elements may indicate that fusion of the braincase and dermal skull roof occurs earlier than fusion in the orbital region, and that the Albertan skulls represent sub‐adult individuals in which fusion is not complete. This idea that fusion increases with age was also suggested for *V. comptoni* (Brito, 1992) and is supported by the incomplete fusion on the right side of an individual *B. lamarquensis* specimen (Bogan et al., 2011).

The fused endocranium of aspidorhynchids has been described by some authors as including the opisthotic (e.g., Bogan et al., 2011). The opisthotic is an endochondral bone that forms the posterior part of the otic region in cladistian and early actinopterygian fishes (Patterson, 1982). It is present in some halecomorphs and primitive teleosts but it is absent in amiiforms (Grande & Bemis, 1998). Because the elements of the braincase in the Albertan crania are indistinguishably fused, we cannot determine if an opisthotic was present or absent in these skulls.

Fusion between the first vertebra and basioccipital is considered to be a feature of all aspidorhynchids (Bogan et al., 2011). However, the first vertebra is not fused to the posterior basioccipital facet in the Albertan crania. The well‐preserved posterior basioccipital facet shows no evidence in the fossil or in the μ‐CT scans that a centrum is attached. In addition, the fused exoccipitals are clearly identifiable, and extend posteriorly to the same extent as the basioccipital facet, again indicating that the first vertebra is not in articulation with the basioccipital.

### 4.2 Taxonomic placement of the aspidorhynchid crania

The pattern of ornamentation and fusion of the dermal skull roof, as well as the fusion of the endocranial elements, confirm that the two Albertan specimens represent an aspidorhychid fish. According to Brito and Suárez (2003), *Aspidorhynchus* species lack ganoine on the skull bones, and, therefore, this is not a candidate genus for the Albertan crania. Dermal skull bones are unknown for *Pseudovinctifer*, but this fish has only been reported from South America (Arratia, 2015), making it less likely to be represented by the Albertan material. Similarly, although the other three genera have ganoine on the skull bones, *Richmondichthys* is known only from Australia (Bartholomai, 2004), and *Jonoichthys* and *Vinctifer* are known only from South America (Brito, 1997; Gouiric‐Cavalli, 2015) making these genera also less likely geographically as candidates for the Albertan neurocrania. Consequently, the two crania likely belong to *Belonostomus*. The majority of *Belonostomus* species are diagnosed based on the morphology of their jaws, scales, and postcrania (e.g., Brito, 1997; Ebert, 2014). This is problematic for the two Albertan crania because they are not associated with any rostral fragments, scales, or postcranial material. *B. longirostris* is the only aspidorhynchid currently known from Alberta, and it is from the same formation as the crania. However, in the absence of overlapping material, we hesitate to assign the crania to this species because examination of aspidorhynchid jaw fragments from the Dinosaur Park Formation indicate to us that there may be more than one species represented.

Further complicating assignment of the crania to *B. longirostris* is that all the material known from the Campanian Dinosaur Park Formation, including the holotype, is fragmentary and cannot unambiguously be assigned to the genus *Belonostomus* because none of it allows an assessment of the diagnostic characters of the genus. The generic diagnosis depends on characters such as relative length of the predentary and premaxillary bones (see Bogan et al., 2011; Brito, 1997) which cannot be determined in disarticulated fragmentary specimens. In favor of this generic assignment for the two crania is that *Belonostomus* is a wide‐spread aspidorhynchid taxon that is found in mid to Late Cretaceous deposits. *Aspidorhynchus*, and *Jonoichthys* are only known from Jurassic deposits (Brito & Suárez, 2003; Gouiric‐Cavalli, 2015), and both *Richmondichthys* from Australia and *Pseudovinctifer* from Chile are Early Cretaceous in age (Arratia, 2015; Bartholomai, 2004). Only *Vinctifer* has also been reported in Cretaceous deposits of North America (in Mexico), but it is a more southern genus, with most fossils, including the Late Cretaceous ones, known from South America (Brito, 1997).

Because of the ornamentation pattern of the dermal skull roof and ganoine covering, and the geographic distribution and age of the Albertan crania, we here consider them likely to belong to the genus *Belonostomus*, but refrain from assigning them to *B. longirostris*. We also suggest that material from Chile assigned to *B. longirostris* by Brito and Suárez (2003) might need to be removed from that species. This is because material from the type locality in Alberta (Figure 1d) shows that the fused premaxillae have a single left and right tooth row which merge together and end in advance of the tip of the fused premaxillae. In the Chilean specimen, the left and right tooth rows of the premaxillae remain separate along their lengths and extend to the tip of the premaxilla (Brito & Suárez, 2003: pl. 1, fig. A). The separate tooth rows are also found in another species of *Belonostomus*, *Belonostomus aciculiferus* from Uzbekistan (Brinkman et al., 2025), indicating this feature may be more broadly distributed among species. The genus *Belonostomus* as currently constituted has a very broad geographic range in the Late Cretaceous. It is found throughout the Western Interior Seaway of North America and Pacific coastal areas of South America (Brito & Suárez, 2003), and in freshwater deposits of Uzbekistan (Brinkman et al., 2025), ranging from Albian (Chile) through Turonian (Uzbekistan) to Cenomanian in age.

### 4.3 Aspidorhynchiform–teleost relationship

Aspidorhynchidae has been considered by some authors to belong to Teleostei (e.g., Brito, 1999; Patterson, 1973), whereas other authors place the family outside Teleostei (e.g., Arratia, 2001; Taverne, 1998). The teleost relationships of aspidorhynchids are not supported here based on the absence of the posterior myodome, and the separation between the trigemino‐facialis chamber and a canal likely for passage of the jugular vein.

The myodome is a median chamber or channel ventral to the braincase through which the external and internal rectus muscles of the eye extend (Allis Jr., 1918). The anterior part of the myodome, called the anterior myodome, forms in the prootic region (Allis Jr., 1918). The anterior myodome is present in aspidorhynchids (see also Brito, 1992). In the Albertan crania, it is a median short canal, and not a chamber with dorsal and ventral compartments as found in most teleosts (Allis, Jr., 1918). It is limited to the prootic as in holosteans (Allis, 1918). A posterior extension of this canal into the basioccipital is referred to as the posterior myodome. The posterior myodome is floored by the parasphenoid and roofed by the basioccipital (Allis Jr., 1918). Although the posterior myodome has been lost in a few teleost lineages (e.g., catfish; Allis Jr., 1918), it is not present at all in pre‐teleost lineages, and its presence is considered to be a synapomorphy for Teleostei (e.g., Patterson, 1973; Pinna, 1996).

Within Aspidorhynchidae, a posterior myodome was reported as present in *Richmondichthys* (Bartholomai, 2004). However, based on the anatomy presented in that study, the myodome described is actually the anterior myodome, because it is reported as being separated into left and right halves by the basisphenoid, and having its posterior extent formed by the prootic (Bartholomai, 2004, pp. 525–526); therefore, it does not extend into the basioccipital and is not a posterior myodome. We conclude that a posterior myodome is not present in *Richmondichthys*.

A posterior myodome was also reported as present in the paratype of *B. lamarquensis* (Bogan et al., 2011). In this case, the authors indicated that although the basioccipital of this specimen is lost, the posterior end of the myodome chamber is visible just below the foramen magnum and, therefore, must have entered the basioccipital. The accompanying drawing (Bogan et al., 2011: fig. 7) shows an opening dorsal to the parasphenoid in this region. However, there is no such opening in the same region in the Albertan specimens (Figure 8). Preliminary re‐examination of this specimen by the original describer indicates this specimen does not have a true posterior myodome (S. Bogun, personal communication, November, 2025). In the Albertan crania, the ventral surface of the posterior skull is concave, but this concavity is in the ventral surface of the parasphenoid, not the basioccipital, and so it does not fit Allis' (1918) definition of the posterior myodome. It also does not connect with the anterior myodome in these crania. The anterior myodome is clearly present in these crania, located anteriorly, surrounded by the sphenotic region, and with the parasphenoid forming the floor of the cavity (Figure 8a). The anterior myodome ends in the anterior area of the prootic. Posterior to this, the parasphenoid becomes laterally compressed and there is no chamber present (Figure 8b). The basioccipital has no ventral chamber or channel in any part of its length. Therefore, there is no posterior myodome in the Albertan aspidorhynchid.

Teleost relationships of aspidorhynchids are also unlikely based on the form of the trigemino‐facialis chamber in the Albertan crania. The trigemino‐facialis chamber in fishes is located in the lateral wall of the neurocranium. In teleosts, this chamber in the prootic is subdivided into a more medially positioned channel for the ganglia of the profundus, trigeminal, and facial nerves, termed the pars ganglionaris, and a horizontal, more lateral subdivision called the pars jugularis for passage of several nerves (trigeminal, profundus, and three branches of the facial) as well as the jugular vein and orbital artery (e.g., Patterson, 1964). In the Albertan fossil neurocrania, there is a large horizontal canal passing through the sphenotic/prootic region just posterior to the orbit (Figures 7 and 8a), ventral to the orbital opening for the trigemino‐facialis chamber. This canal does not connect with the cranial cavity, so we believe it likely to be for the circulatory system, probably the jugular vein. This indicates that the trigemino‐facialis chamber in the Albertan aspidorhynchid does not have a teleost morphology.

The inclusion of Aspidorhynchidae within Teleostei has been a matter of disagreement among researchers. Aspidorhynchids have been positioned as an early diverging lineage of teleosts by Patterson (1973), Pinna (1996), and Brito (1999). The family has been excluded from Teleostei by Taverne (1998) and Arratia (2001), the latter of whom suggested reserving the name Teleostei for the “true” teleosts based on presence of apomorphies, and using the name Teleosteomorpha for the true teleosts along with the many fossil stem groups. The lack of the posterior myodome in the Albertan aspidorhynchid and non‐teleost morphology of the trigemino‐facialis chamber indicates that Aspidorhynchidae is not a member of Teleostei. The contradiction between these specimens and the possible presence of the posterior myodome in *B. lamarquensis* suggests that the paratype specimen of that species showing the feature might benefit from a re‐examination using μ‐CT scanning.

## 5 CONCLUSIONS

The two crania from the Dinosaur Park Formation of Alberta are contemporaneous and from the same formation as *B. longirostris*. They likely belong to a species of *Belonostomus*, based on ornamentation pattern and locality data, but the material does not overlap with the holotype (a partial jaw) of *B. longirostris* and there may be more than one species of *Belonostomus* in this formation (personal observation DBB, November 2025). We here identify the crania only to the genus *Belonostomus*. New information from these crania and the μ‐CT scans document that the first vertebra is not sutured to the basioccipital, the posterior myodome is not present, and there is a separate canal, presumably for the jugular, that does not communicate with the trigemino‐facialis chamber. These features indicate aspidorhynchids are not teleost fishes. Additionally, fusion among elements of the endochondral braincase and among elements of the dermal skull roof is complete, but there is no fusion among the bones in the orbital region (orbitosphenoid and pterosphenoids). This might indicate that these crania are from sub‐adult individuals and the fusion of elements progresses from posterior to anterior in the cranial region.

## AUTHOR CONTRIBUTIONS

**Mondo Miyazato:** Formal analysis; investigation; visualization; writing – original draft; writing – review and editing. **Luke E. Nelson:** Formal analysis; investigation; visualization; writing – review and editing. **Joshua A. Doyon:** Formal analysis; investigation; visualization; writing – review and editing. **Donald B. Brinkman:** Conceptualization; investigation; visualization; writing – review and editing. **Alison M. Murray:** Conceptualization; investigation; visualization; supervision; writing – original draft; writing – review and editing; funding acquisition.

## CONFLICT OF INTEREST STATEMENT

The authors declare that they have no competing interests.

## Data Availability

All fossil specimens described in this study are cataloged and deposited in the Royal Tyrrell Museum of Palaeontology (Drumheller, Canada), a publicly accessible museum. CT images and segmented 3D models are accessible from the University of Alberta Educational and Research Archive (ERA) https://ualberta.scholaris.ca/home (Belonostomus); original specimens are permanently curated in the collections of the Royal Tyrrell Museum of Palaeontology.
